# Renal Overexpression of Atrial Natriuretic Peptide and Hypoxia Inducible Factor-1**α** as Adaptive Response to a High Salt Diet

**DOI:** 10.1155/2014/936978

**Published:** 2014-02-13

**Authors:** Silvana Lorena Della Penna, Gabriel Cao, Andrea Carranza, Elsa Zotta, Susana Gorzalczany, Carolina Susana Cerrudo, Natalia Lucía Rukavina Mikusic, Alicia Correa, Verónica Trida, Jorge Eduardo Toblli, María Inés Rosón, Belisario Enrique Fernández

**Affiliations:** ^1^Department of Pathophysiology, School of Pharmacy and Biochemistry, University of Buenos Aires, INFIBIOC-CONICET, Argentina; ^2^Cátedra de Fisiopatología, Facultad de Farmacia y Bioquímica, UBA, Junín 956, Piso 5, 1113 Buenos Aires, Argentina; ^3^Laboratory of Experimental Medicine, Hospital Alemán, Buenos Aires, Argentina; ^4^Department of Pharmacology, School of Pharmacy and Biochemistry, University of Buenos Aires, INFIBIOC-CONICET, Argentina; ^5^Department of Clinical Biochemistry, School of Pharmacy and Biochemistry, University of Buenos Aires, INFIBIOC-CONICET, Argentina

## Abstract

In the kidney, a high salt intake favors oxidative stress and hypoxia and causes the development of fibrosis. Both atrial natriuretic peptide (ANP) and hypoxia inducible factor (HIF-1*α*) exert cytoprotective effects. We tested the hypothesis that renal expression of ANP and HIF-1*α* is involved in a mechanism responding to the oxidative stress produced in the kidneys of rats chronically fed a high sodium diet. Sprague-Dawley rats were fed with a normal salt (0.4% NaCl) (NS) or a high salt (8% NaCl) (HS) diet for 3 weeks, with or without the administration of tempol (T), an inhibitor of oxidative stress, in the drinking water. We measured the mean arterial pressure (MAP), glomerular filtration rate (GFR), and urinary sodium excretion (UV_Na_). We evaluated the expression of ANP, HIF-1*α*, and transforming growth factor (TGF-*β*1) in renal tissues by western blot and immunohistochemistry. The animals fed a high salt diet showed increased MAP and UV_Na_ levels and enhanced renal immunostaining of ANP, HIF-1*α*, and TGF-*β*1. The administration of tempol together with the sodium overload increased the natriuresis further and prevented the elevation of blood pressure and the increased expression of ANP, TGF-*β*1, and HIF-1*α* compared to their control. These findings suggest that HIF-1*α* and ANP, synthesized by the kidney, are involved in an adaptive mechanism in response to a sodium overload to prevent or attenuate the deleterious effects of the oxidative stress and the hypoxia on the development of fibrosis.

## 1. Introduction

Reactive oxygen species (ROS) have been demonstrated to play an important pathophysiological role in the kidney [1–4]. ROS can activate the mitochondrial uncoupling protein 2 (UCP-2), leading to inefficient renal O_2_ usage and contributing to renal hypoxia [5]. Changes in cellular oxygen concentrations induce tightly regulated response pathways that attempt to restore oxygen supply to cells and modulate cell function in hypoxic conditions. Most of these responses occur through the induction of the transcription factor hypoxia inducible factor-1 (HIF-1) which coordinates the expression of diverse adaptive genes against the hypoxic injury [6, 7]. HIF-1 transcriptionally upregulates the expression of metabolic proteins (GLUT-1), adhesion proteins (integrins), soluble growth factors (TGF-*β* and VEGF), and extracellular matrix components (type I collagen and fibronectin), which enhance the repair process. For these reasons, HIF-1 is viewed as a positive regulator of organ repair and tissue fibrosis [8, 9].

In the kidney, a predominant enzyme involved in oxidative stress development is NADPH-oxidase, which is upregulated by increased sodium tubular transport, luminal flow, or cytokines release [10, 11]. In turn, the superoxide anion increases tubular NaCl transport, further enhancing oxidative stress [12]. It is well known that a high salt intake increases the oxidative stress in the kidneys of normal and salt-sensitive rats [1–4]. In this regard, we have reported that a high salt diet in Sprague-Dawley rats is able to increase the oxidative stress. We also showed that the administration of tempol (4-hydroxy-2,2,6,6-tetramethylpiperidine-*N*-oxyl), which is a permeate superoxide dismutase mimetic commonly used to inhibit the oxidative stress, prevented those changes and produced a potent natriuretic and diuretic effect. Thus, we concluded that the increase of the oxidative stress induced by sodium overload could account for antinatriuresis [1]. It has also been described that, in models of experimental salt-sensitive hypertension, tempol improved the renal hemodynamic response and electrolyte excretory function, while abolishing salt-sensitive hypertension and renal oxidative stress [13].

A growing number of mammalian genes have been identified to play a key role in the cellular adaptive response to counter-regulate the renal hypoxia and the consequent process of fibrosis, among which is atrial natriuretic peptide (ANP). ANP is a member of a natriuretic peptide family which, besides its role in the regulation of volume homeostasis, has been noted to exert protective effects in several cell types in response to oxidative stress [14] and fibrosis [15] and on the adaptation to hypoxia [16].

Based on these data, we hypothesized that endogenous renal ANP and HIF-1*α* could constitute endogenous adaptive mechanisms in response to the oxidative stress produced in the kidney of rats chronically fed with a high sodium diet. Therefore, in the current study we determined the effect of a high-salt diet on the regulation of renal expression of HIF-1*α*, ANP, and TGF-*β*1 in Sprague-Dawley rats. In addition, we evaluated whether tempol administration, by inhibiting oxidative stress, prevents the increase of these molecules. Understanding these endogenous mechanisms can lead to finding a better therapeutic approach in salt-sensitive hypertension.

## 2. Methods

### 2.1. Animal Protocol and Experimental Measurements

Experiments were conducted in accordance with the care and use of research animals of international guiding principles and local regulations concerning the care and use of laboratory animals for biomedical research (ANMAT, 6344/96; Institute of Laboratory Animal Resources, 1996) (14), as well as the “International Ethical Guiding Principles for Biomedical Research on Animals” established by the CIOMS (Council for International Organizations of Medical Sciences) (15). These protocols were approved by Universidad de Buenos Aires (UBACYT B113) and the National Scientific and Technical Research Council (CONICET, PIP 1337/09).

Male Sprague Dawley rats, 5-6 weeks old (180–200 g body weight), were used in the experiments. The animals were housed in steel cages in a controlled temperature animal room at 23 ± 2°C, exposed to a daily 12-hour light-dark cycle (light on from 07:00 a.m. to 07:00 p.m.), fed the diets described below for three weeks, and were given free access to tap water. The experiments were performed in rats randomly divided into four groups (*n* = 6 for each group): (a) NS (control): fed a normal salt diet (0.4 g% NaCl); (b) HS: fed a high salt diet (8% g NaCl); (c) NS-T: fed a normal salt diet (0.4 g% NaCl) plus 1 mM tempol (Sigma-Aldrich Inc, St. Louis, Missouri, USA), administered in the drinking water; (d) HS-T: fed a high salt diet (8% g NaCl) plus 1 mM tempol administered in the drinking water. After 3 weeks, the rats were anaesthetized intraperitoneally with urethane (1.2 g·kg^−1^) and a PE-90 tube (3 cm long) was inserted into the trachea to maintain an open airway. The left femoral vein was catheterized with a Silastic cannula (0.12 mm i.d.) for continuous infusion. The right carotid artery was catheterized with a T4 tube for blood sampling and for continuous mean arterial pressure recording (MAP) by means of a Statham GOULD P23ID transducer coupled to a Grass Polygraph 79D during all the procedures. The bladder was cannulated for urine collection using a PE-75 cannula. A femoral vein infusion with isotonic saline solution (ISS, 0.15 M NaCl) was performed at a rate of 0.04 mL·min^−1^ (Syringe Infusion Pump, Sage, Orion) for 60 minutes to allow for a steady diuresis and to permit urine collection in all groups. Then, ISS infusion continued for another 60 min at the same rate during the experimental period. From each animal, a blood sample was collected at 30 minutes and a urine sample was collected from 0 to 60 minutes for sodium, potassium, and creatinine measurements. At the end of the experimental period, other blood samples were obtained from the abdominal cava vein, immediately placed into plastic tubes containing 15% EDTA, and kept on ice for ANP dosage. The kidney was rapidly excised, decapsulated, longitudinally cut, and harvested for immunohistochemical studies.

### 2.2. ANP Radioimmunoassay

The plasma ANP extraction procedure was followed as described by Cavallero et al. [17]. The radioimmunoassay (RIA) was performed using an ANP-rat RIA commercial kit (Phoenix Pharmaceuticals, Burlingame, CA) [18].

### 2.3. Urine and Blood Measurements

Urinary and plasma sodium and creatinine were measured by standard methods using an autoanalyzer. Creatinine clearance was assessed in order to evaluate the glomerular filtration rate (GFR). GFR and sodium fractional excretion (FE_Na_) were calculated according to a standard formula. Urinary flow (UV) is expressed as *μ*L·min^−1^, plasmatic sodium (PL_Na_) concentration as mEq·L^−1^, sodium urinary excretion (UV_Na_) as *μ*mol·min^−1^, GFR as mL·min^−1^, and FE_Na_ as percentage.

### 2.4. Kidney Processing for Histological Examination

Renal tissues were fixed in phosphate-buffered 10% formaldehyde (pH 7.20) and embedded in paraffin. For immunohistochemistry, sections were deparaffinated and rehydrated, and endogenous peroxidase activity was blocked by treatment with 0.5% H_2_O_2_ in methanol for 20 minutes. Local ANP, HIF-1*α*, and TGF-*β*1 were detected using the following specific antibodies: rabbit anti-ANP (Phoenix Pharmaceutical; dilution 1 : 500), rabbit anti-HIF-1*α* (Novus Biologicals, Inc., Littleton, CO; dilution 1 : 1000), and rabbit anti-TGF-*β*1 (Santa Cruz Biotechnology; dilution 1 : 200). Immunostaining was performed with a commercial modified avidin-biotin-peroxidase complex technique (Vectastain ABC kit, Universal Elite, Vector Laboratories, CA) and with counterstaining with haematoxylin. Histological sections were observed in a Nikon E400 light microscope (Nikon Instrument Group, Melville, New York, USA). All measurements were carried out using image analysis software (Image-Pro Plus ver. 4.5 for Windows, Media Cybernetics, LP. Silver Spring, MD, USA). Immunoreactivities for ANP, HIF-1*α*, and TGF-*β*1 are expressed as percentage of positive stained area ± standard error of the mean (SEM) in proximal convoluted tubules (PT), distal tubules (DT), thick ascending limb of the loop of Henle (THAL), cortical collecting ducts (CCD), and medullary collecting ducts (MCD). Tissue samples of all the animals were evaluated in a blinded fashion and separately by two researchers regarding knowledge of treatments.

#### 2.4.1. Preparation of Renal Homogenates for Western Blot

The right kidney from all groups was extracted, dissected, and separated. Tissue samples were homogenized on ice with a Tissue Tearor (Biospec Products Inc.) in a buffer mixture (50 mmol·L^−1^ Tris, 0.1 mmol·L^−1^ EDTA, 0.1 mmol·L^−1^ EGTA, 1% Triton, 1 mmol·L^−1^ PMSF, 1 *μ*mol·L^−1^ pepstatin, 2 *μ*mol·L^−1^ leupeptin, and 1x protease inhibitor cocktail (Roche Diagnostics). Protein concentration in the Triton-soluble supernatant was determined by the Lowry technique.

#### 2.4.2. Western Blot Analysis for HIF-1*α* and ANP

Pooled samples of renal tissue from five animals of each group and containing similar amounts of protein (150 *μ*g protein/lane) were separated by electrophoresis in 10% SDS polyacrylamide gels (Bio-Rad) and then transferred to a nitrocellulose membrane (Bio-Rad) and incubated with mouse polyclonal anti-ANP (Santa Cruz Biotechnology, Inc. 1 : 800 dilution) or rabbit monoclonal-anti-HIF-1*α* (Abcam; 1 : 1000 dilution). For ANP, a secondary immune reaction followed with a goat anti-rabbit IgG (H + L) conjugated with horseradish peroxidase (dilution of 1 : 10000). For HIF-1*α* a secondary immunereaction followed with a biotinylated anti-rabbit (dilution of 1 : 1000) and a 3rd antibody conjugated with horseradish peroxidase (dilution of 1 : 1000). The samples were revealed by chemiluminescence using ECL reagent (Amersham Pharmacia Biotech) for 2–4 min. The density of the respective bands was quantified by densitometric scanning of Western blots using a Hewlett-Packard scanner and Totallab analyzer software (Biodynamics Corp.). To avoid inaccuracies in protein loading, beta-tubulin was measured as internal standard (anti-beta tubulin, Ab 6046, rabbit polyclonal antibody) for each blot and protein levels were calculated and expressed as the ratio between the optical densities of the bands corresponding to ANP or HIF-1*α* and *β*-tubulin.

### 2.5. Statistical Analysis

Results from urine and blood measurements and MAP are expressed as mean ± SEM. Gaussian distribution was evaluated by the Kolmogorov and Smirnov method and the comparison among groups was carried out using ANOVA followed by the Bonferroni test. *P*  values < 0.05 were considered significant.

## 3. Results

### 3.1. Body Weight and Mean Arterial Pressure

There were no significant differences in body weight between control and experimental groups (grams; NS: 330 ± 9; HS: 320 ± 6; NS-T: 314 ± 13; HS-T: 320 ± 7).

The MAP was increased in the HS fed group compared to the NS-fed group (mmHg; NS: 94 ± 3 versus HS: 107 ± 3*). Tempol administration did not modify MAP levels in NS animals but normalized MAP in the HS-T group, reaching MAP levels very similar to those observed in NS rats (mmHg; NS-T: 97 ± 2 versus HS-T: 95 ± 3^†^) *P < 0.05  versus respective NS group, ^†^ *P* < 0.05 versus respective group without tempol.

### 3.2. Plasma Sodium and Urinary Sodium Excretion (Table 1)

High salt diet and tempol administration did not alter plasma sodium in any experimental group.

The administration of a high salt diet did not alter the GFR, which was increased by tempol in HS-T group with respect to the HS group. Tempol administration also increased GFR in the NS-T group as compared with the NS group.

The administration of a high salt diet led to a greater UV_Na_, which was further increased in HS-T group with respect to the NS-T and HS groups. Moreover, tempol increased UV_Na_ in NS fed rats.

The administration of a high salt diet raised FE_Na_ in HS group with respect to the NS group. Tempol administration increased the FE_Na_ further in the HS-T group as compared with NS-T and to the HS group. FE_Na_ was not altered in the NS-T group.

### 3.3. Plasmatic ANP

Plasma ANP concentration (pg·mL^−1^) did not differ significantly between the NS (325.75 ± 66.69) and HS (260.00 ± 7.38) groups, as measured by RIA.

### 3.4. Intrarenal ANP Expression

**Figure 1:**
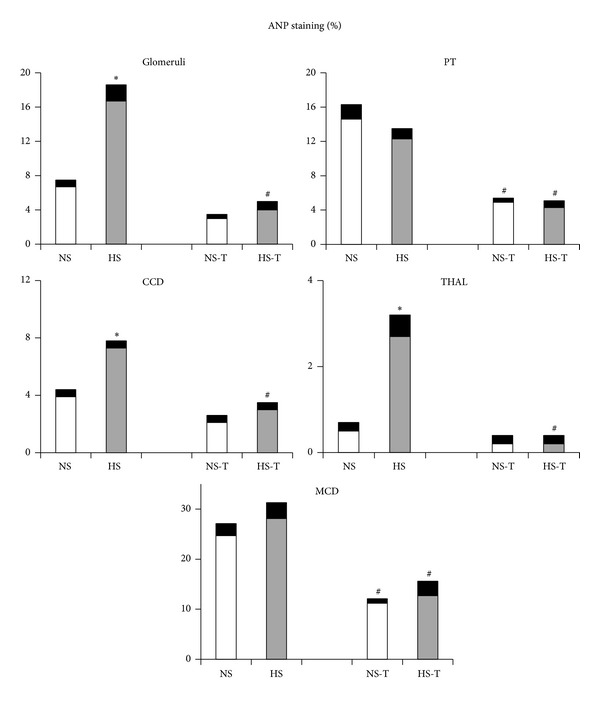
Quantitative representation of positive intrarenal atrial natriuretic peptide (ANP) immunostaining/*μ*m^2^. Values are expressed as percentage ± SEM, *n* = 5-6, *P < 0.05 versus respective NS control; ^#^ *P* < 0.05 versus respective control without tempol. NS: normal salt diet group, HS: high salt diet group, NS-T: normal salt diet plus tempol group, HS-T: high salt diet plus tempol group. PT: proximal tubule, CCD: cortical collecting duct, THAL: thick ascending limb of the loop of Henle, and MCD: medullary collecting duct.

**Figure 2:**
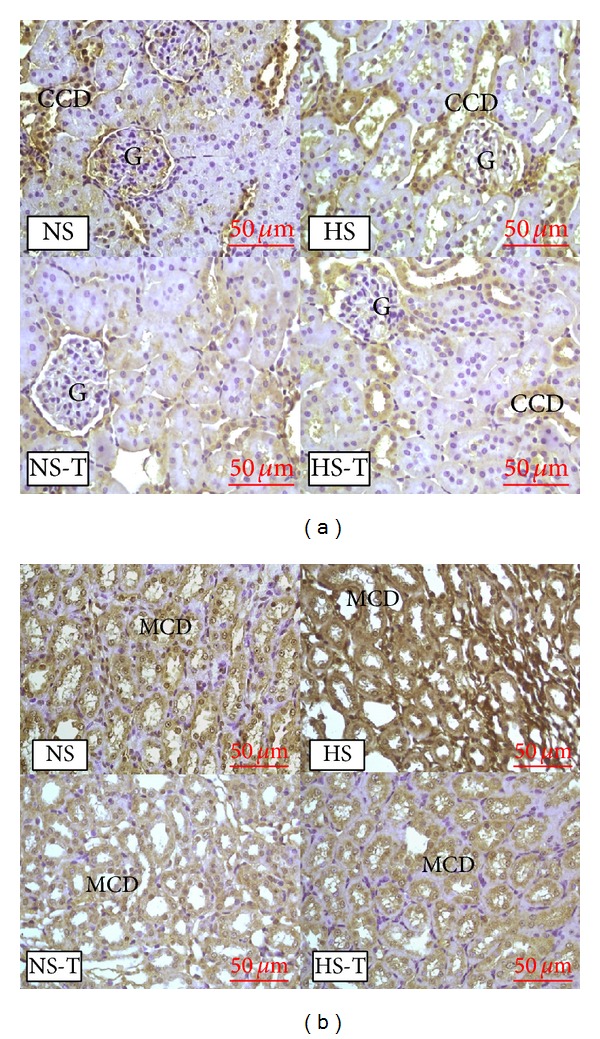
(a) Representative images of positive staining of atrial natriuretic peptide (ANP) in renal cortex. (b) Representative images of positive staining of ANP in renal medulla. Original magnification 400x. NS: normal salt diet group, HS: high salt diet group, NS-T: normal salt diet plus tempol group, HS-T: high salt diet plus tempol group. G: glomerulus, CCD: cortical collecting duct, MCD: medullary collecting duct.

Figure 1 shows quantified levels of ANP immunoexpression in renal tissues. Representative images of ANP positive staining are shown in Figure 2(a) renal cortex and Figure 2(b) renal medulla. The analysis of renal sections obtained from the HS group revealed increased positive staining for ANP in glomeruli, THAL, and CCD with respect to the NS group. Tempol prevented the elevation of ANP staining in HS-T group in glomeruli, THAL, and CCD and decreased ANP staining in PT and MCD of both NS-T and HS-T groups.

**Figure 3:**
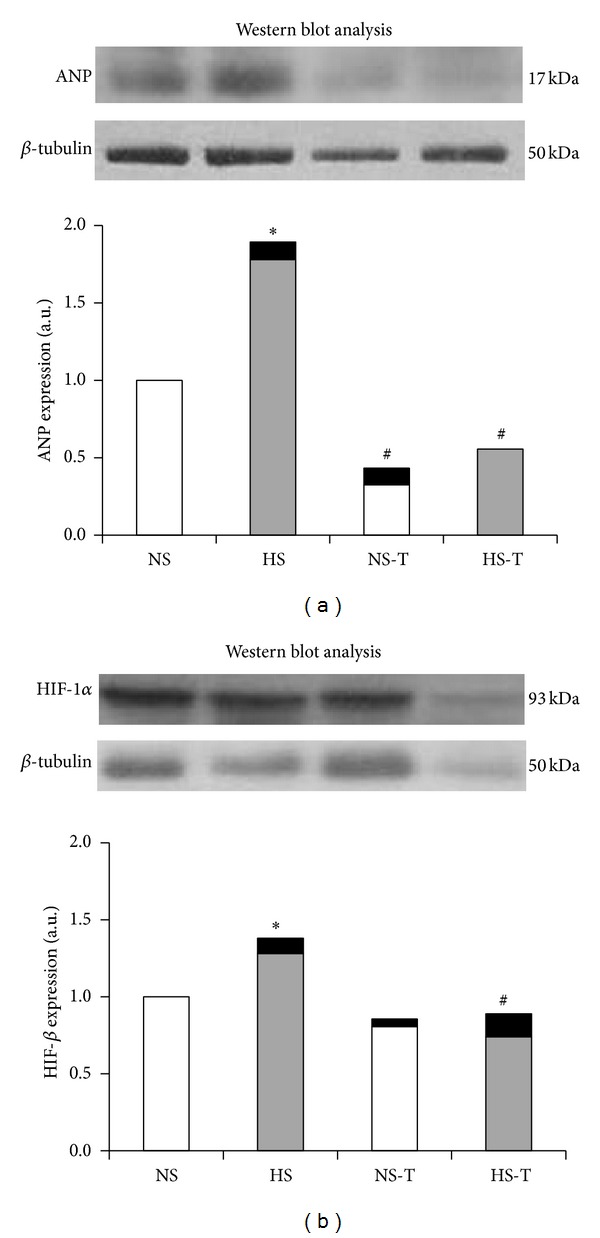
Representative Western blot analysis in renal tissue of ANP (a) and HIF-1*α* (b). NS: normal salt diet group, HS: high salt diet group, NS-T: normal salt diet plus tempol group, and HS-T: high salt diet plus tempol group. Histograms illustrate the values of protein expression of ANP and HIF-1*α* for every group. Each blot was normalized to the expression of *β*-tubulin from the same gel. Data are mean ± SEM, expressed as arbitrary units. *n* = 5-6. *P < 0.05 versus NS group; ^#^ *P* < 0.05 versus respective group without tempol.

Figure 3(a) shows quantified protein expression of ANP in renal tissue by Western blot analysis. The analysis revealed increased positive staining of HS group for ANP. Tempol decreased the expression of both NS-T and HS-T groups compared to their respective control without tempol.

### 3.5. Intrarenal HIF-1*α* Expression

**Figure 4:**
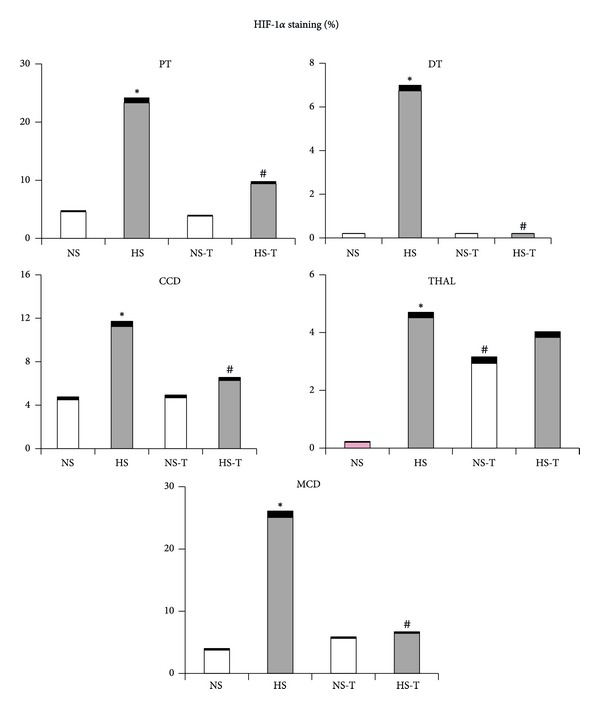
Quantitative representation of positive intrarenal hypoxia inducible factor-1 (HIF-1*α*) immunostaining/*μ*m^2^. Values are expressed as percentage ± SEM. *n* = 5-6. *P < 0.05 versus respective NS control; ^#^ *P* < 0.05 versus respective control without tempol. NS: normal salt diet group, HS: high salt diet group, NS-T: normal salt diet plus tempol group, HS-T: high salt diet plus tempol group. PT: proximal tubule, DT: distal tubule, CCD: cortical collecting duct, THAL: thick ascending limb of the loop of Henle, and MCD: medullary collecting duct.

**Figure 5:**
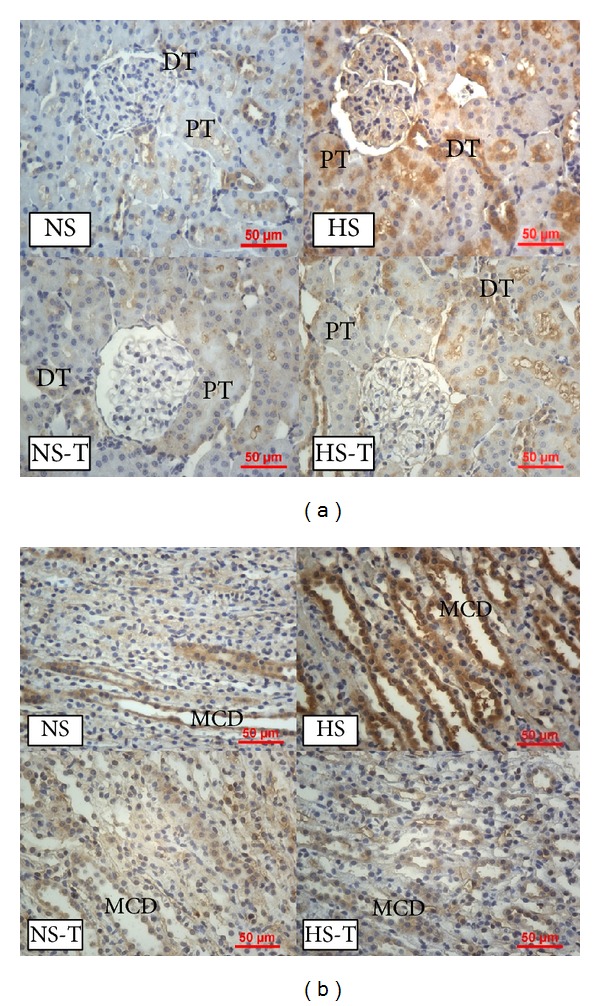
(a) Representative images of positive staining of hypoxia inducible factor-1 (HIF-1*α*) in renal cortex. (b) Representative images of positive staining of HIF-1*α* in renal medulla. Original magnification 400x. NS: normal salt diet group, HS: high salt diet group, NS-T: normal salt diet plus tempol group, HS-T: high salt diet plus tempol group. PT: proximal tubule, DT: distal tubule, and MCD: medullary collecting duct.

Figure 4 shows quantified levels of HIF-1*α* immunoexpression in renal tissues. Representative images of HIF-1*α* positive staining are shown in Figure 5(a) renal cortex and Figure 5(b) renal medulla. Immunostaining analysis of renal sections obtained from HS group revealed that HIF-1*α* staining was increased in all examined tubular segments (PT, DT, CCD, THAL, and MCD) with respect to the NS group. The administration of tempol did not alter HIF-1*α* staining in the NS-T group, except in THAL where it was augmented. Whereas in HS-T group, HIF-1*α* staining decreased in all tubular segments except in THAL compared with HS group.

The quantified protein expression of HIF-1*α* in renal tissue by Western blot analysis can be observed in Figure 3(b). The analysis revealed increased positive staining of HS group for HIF-1*α*. Tempol decreased the expression in HS-T groups compared to its control without tempol.

### 3.6. Intrarenal TGF-*β*1 Expression

**Figure 6:**
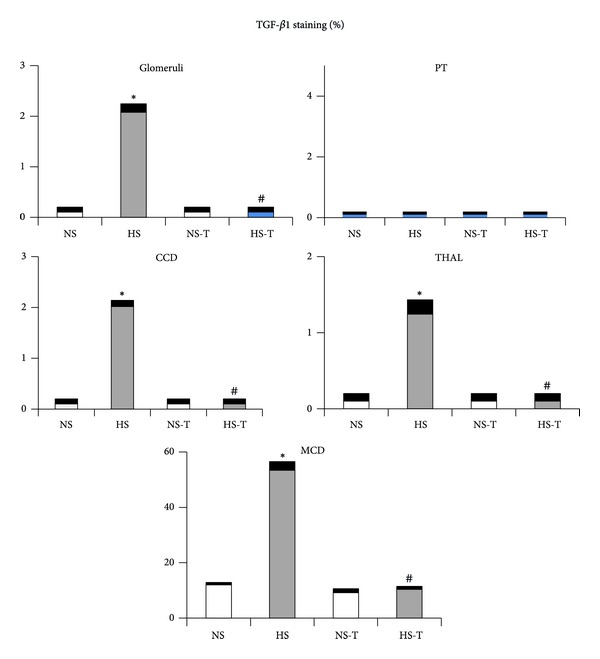
Quantitative representation of positive intrarenal transforming growth factor (TGF-*β*1) immunostaining/*μ*m^2^. Values are expressed as percentage ± SEM, *n* = 5-6. *P < 0.05 versus respective control NS; ^#^ *P* < 0.05 versus respective control without tempol. NS: normal salt diet group, HS: high salt diet group, NS-T: normal salt diet plus tempol group, HS-T: high salt diet plus tempol group, PT: proximal tubule, CCD: cortical collecting duct, THAL: thick ascending limb of the loop of Henle, and MCD: medullary collecting duct.

**Figure 7:**
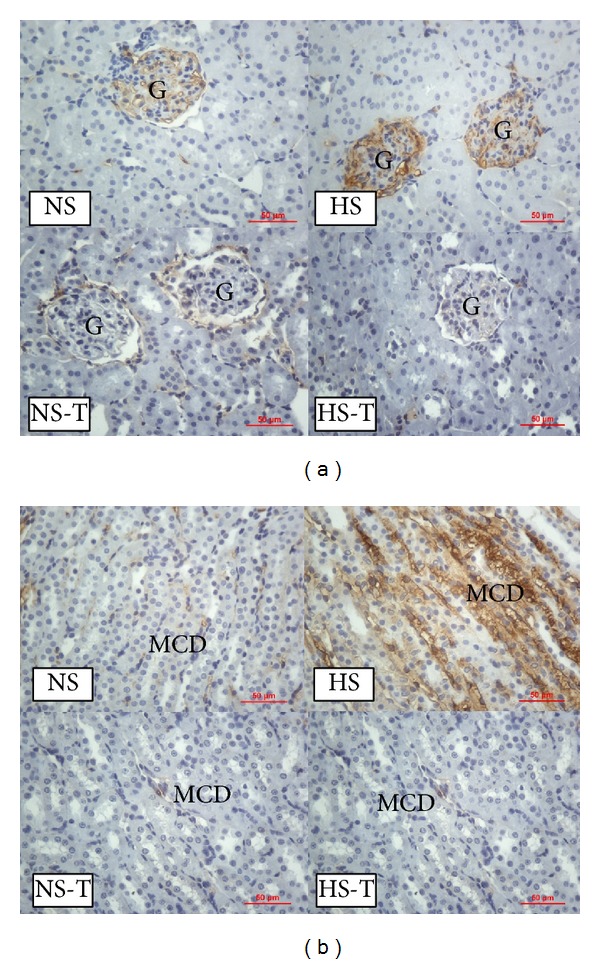
(a) Representative images of positive staining of TGF-*β*1 in renal cortex. (b) Representative images of positive staining of TGF-*β*1 in renal medulla. Original magnification 400x. NS: normal salt diet group, HS: high salt diet group, NS-T: normal salt diet plus tempol group, HS-T: high salt diet plus tempol group, G: glomerulus, and MCD: medullary collecting duct.

Figure 6 shows quantified levels of TGF-*β*1 immunoexpression in renal tissues. Representative images of TGF-*β*1 positive staining are shown in Figure 7 in (a) renal cortex and (b) renal medulla. Immunostaining analysis of renal sections revealed increased positive staining for TGF-*β*1 in glomeruli, THAL, CCD, and the MCD in HS group, with respect to the NS group. Tempol did not show additional effects in the NS-T group as compared with its control and prevented the increase of TGF-*β*1 staining in HS-T group.

## 4. Discussion

Our results showed that a high salt diet in normal rats resulted in a greater ANP expression in glomeruli, THAL and CCD, where the profibrotic marker TGF-*β*1 was also increased. In addition, enhanced HIF-1*α* expression was observed not only in the renal medulla as it has been previously described [19], but also in renal cortex. We found that tempol administration, a superoxide dismutase mimetic, favored urinary sodium excretion, prevented the increase of ANP and TGF-*β*1 expression, and normalized HIF-1*α* expression, the latter except in THAL. Western blot analysis confirmed our data, showing increased positive staining of HS group for ANP and HIF-1*β* and decreased the expression of tempol groups compared to their respective controls. These results suggest that increased ANP and HIF-1*α* expression in renal cortex and medulla could be involved in an adaptive response to the oxidative stress resulting from a high salt diet.

As we have previously reported, a chronic salt overload (8% NaCl diet during 3 weeks) does not alter body weight or plasma sodium but causes higher MAP levels as well as urinary sodium excretion [1]. Even though the animals used in this study have their blood pressure taken under anesthesia, we have recently reported the same effect of a high salt diet on the MAP in conscious animals [20], allowing us to conclude that the anesthesia does not affect the blood pressure when the different diet groups are compared. On the other hand, the administration of tempol normalized MAP levels observed in the HS group, and it also increased further urinary sodium excretion. These results are in agreement with previous studies, where treatment with tempol improved renal hemodynamic and electrolyte excretory function in salt-sensitive hypertension models [1, 21]. The effects of tempol, promoting natriuresis and preventing endogenous increase of ANP, could suggest that, by inhibiting oxidative stress, tubular sodium transport decreases and thus prevents the endogenous ANP expression as natriuretic hormone. However, we observed a clear raise in ANP expression also in glomeruli from rats fed a high salt diet, in which sodium transport does not occur. Therefore, in addition to a natriuretic effect, endogenous ANP could be exerting other effects in glomeruli and tubules. Furthermore, the increase of renal ANP levels coincided with TGF-*β*1 over-expression in glomeruli, THAL and CCD. In this regard, it is known that TGF-*β*1 upregulates the transcription of the serum and glucocorticoid-dependent kinase hSGK1, involved in the regulation of two important factors for cell volume regulation, that is, the renal epithelial Na^+^ channel ENaC and the thick ascending limb Na^+^, K^+^, and 2Cl^−^ cotransporter NKCC [22]. The increase of cell volume stimulates protein synthesis and inhibits protein degradation, contributing to enhancing the net formation and deposition of matrix proteins. In addition, TGF-*β*1 transduces intracellular signals through type 1 (TGF-*β*R1) and type 2 (TGF-*β*R2) receptors, via the nuclear translocation of Smad3 proteins, thus contributing to a fibrotic response [23]. It has been demonstrated that the activation of ANP/cGMP/PKG signaling phosphorylates Smad3 and disrupts TGF-*β*1-induced nuclear translocation of pSmad3 and later downstream events, including myofibroblast transformation and the proliferation and expression of extracellular matrix molecules [24, 25]. Moreover, we have previously demonstrated that an increase in TGF-*β*1 expression produced by an acute saline overload was prevented and reversed by the administration of low and nonhypotensive doses of ANP [26]. Taking this into account, the present results suggest that the raise of ANP expression in rats fed a high salt diet could constitute a counter-regulatory mechanism against antinatriuretic and/or profibrotic TGF-*β*1 actions.

Meanwhile, the plasmatic concentration of ANP was not affected by dietary salt, suggesting that these animals did not change cardiac ANP secretion [17]. Several reports provide evidence that plasmatic ANP increases after a chronic salt loading given by drinking 1–18% NaCl solutions [27, 28] or by a rat chow with high salt content [29]. However, the literature also shows conflicting results reporting that ANP mRNA expression and circulating levels of ANP remained unaltered after the ingestion of high sodium diets [30]. The present results show that renal ANP expression levels are independent of circulating ANP levels and are subjected to a different regulation. These findings are in accordance with Sun et al. who have previously demonstrated that a dietary salt supplementation may selectively increase ANP levels in the kidney by downregulating its clearance receptor (NPR-C) [31].

Our study also shows that a high salt intake increased HIF-1*α* expression not only in the renal medulla, as it has been described before [18], but also in renal cortex. Considering that a high salt intake increases oxidative stress, renal tubular transport, and hypoxia, it may constitute one of the possible factors involved in the upregulation of HIF-1*α* expression in renal tissues [6, 32]. Additionally, it has been reported that a high salt intake inhibits PHD-2 expression, the predominant isoform in renal medulla of prolyl-hydroxylase enzyme, which promotes the degradation of HIF-1*α*, increasing its expression level [33]. In this way, recent studies have shown that HIF-1*α* may regulate the encoding genes of some enzymes in the THAL, as those of nitric oxide synthase (NOS), cyclooxygenase-2 (COX-2), and hemeoxygenase-1 (HO-1), which are highly expressed in renal medulla [34, 35]. However, the tempol administration, which prevents oxidative stress and enhanced urinary sodium excretion, normalized HIF-1*α* expression in the HS group, except in THAL from both groups fed a normal or a high salt diet. These data suggest that tempol could prevent the hypoxia produced by the oxidative stress as it has been described [5, 20], but not the hypoxia produced by greater sodium transport in THAL, where the enhancement of sodium reabsorption in this tubular segment may be compensating the inhibition of sodium transport in PT, elicited by tempol.

In summary, the present findings suggest that HIF-1*α* and ANP could represent a main adaptive mechanism in normal rats, in response to a salt overload, counter-regulating the hypoxia and fibrosis produced by oxidative stress, and playing a crucial role in the maintenance of sodium balance. The administration of tempol, as a scavenger of superoxide anion, prevents overexpression of *α* and ANP. The disruption of this salt adaptive pathway in salt-sensitive rats could be the cause of sodium retention, oxidative stress, inflammation, and fibrosis in these animals. The pharmacological potentiation of endogenous ANP may be a therapeutic approach for the management of oxidative stress in salt-sensitive hypertension.

## Figures and Tables

**Table 1:** Renal function parameters.

	NS	HS	NS-T	HS-T
PL_Na_ (mEq·L^−1^)	142 ± 1	145 ± 2	143 ± 1	143 ± 2
GFR (mL·min^−1^)	1.51 ± 0.1	1.48 ± 0.1	3.4 ± 0.8^†^	3.1 ± 0.7^†^
UV_Na_ (*μ*mol·min^−1^·kg^−1^)	0.23 ± 0.1	3.50 ± 0.64*	2.00 ± 0.92^†^	13.0 ± 1.5^∗†^
FE_Na_ (%)	0.04 ± 0.02	0.53 ± 0.08*	0.12 ± 0.06	1.20 ± 0.20^∗†^

All values are mean ± SEM, *n* = 5-6, *P < 0.05 versus respective NS group, ^†^ *P* < 0.05 versus respective group without tempol. NS: normal salt diet group, HS: high salt diet group, NS-T: normal salt diet plus tempol group, HS-T: high salt diet plus tempol group, PL_Na_: plasmatic sodium concentration, GFR: glomerular filtration rate, UV_Na_: urinary sodium excretion, and FE_Na_: fractional sodium excretion.

## References

[1] Rosón MI, Della Penna SL, Cao G (2011). High-sodium diet promotes a profibrogenic reaction in normal rat kidneys: effects of tempol administration. *Journal of Nephrology*.

[2] Adler S, Huang H (2004). Oxidant stress in kidneys of spontaneously hypertensive rats involves both oxidase overexpression and loss of extracellular superoxide dismutase. *The American Journal of Physiology*.

[3] Majid DS, Kopkan L (2007). Nitric oxide and superoxide interactions in the kidney and their implication in the development of salt-sensitive hypertension. *Clinical and Experimental Pharmacology and Physiology*.

[4] Feng MG, Dukacz SA, Kline RL (2001). Selective effect of tempol on renal medullary hemodynamics in spontaneously hypertensive rats. *The American Journal of Physiology*.

[5] Lai EY, Luo Z, Onozato ML (2012). Effects of the antioxidant drug tempol on renal oxygenation in mice with reduced renal mass. *The American Journal of Physiology*.

[6] Heyman SN, Rosen S, Rosenberger C (2011). Hypoxia-inducible factors and the prevention of acute organ injury. *Critical Care*.

[7] Schödel J, Klanke B, Weidemann A (2009). HIF-prolyl hydroxylases in the rat kidney: physiologic expression patterns and regulation in acute kidney injury. *American Journal of Pathology*.

[8] Lokmic Z, Musyoka J, Hewitson TD, Darby IA (2012). Hypoxia and hypoxia signaling in tissue repair and fibrosis. *International Review of Cell and Molecular Biology*.

[9] Nie J, Hou FF (2012). Role of reactive oxygen species in the renal fibrosis. *Chinese Medical Journal*.

[10] Garvin JL, Hong NJ (2008). Cellular stretch increases superoxide production in the thick ascending limb. *Hypertension*.

[11] Tian N, Moore RS, Phillips WE (2008). NADPH oxidase contributes to renal damage and dysfunction in Dahl salt-sensitive hypertension. *The American Journal of Physiology*.

[12] Silva GB, Ortiz PA, Hong NJ, Garvin JL (2006). Superoxide stimulates NaCl absorption in the thick ascending limb via activation of protein kinase C. *Hypertension*.

[13] Kobori H, Nishiyama A (2004). Effects of tempol on renal angiotensinogen production in Dahl salt-sensitive rats. *Biochemical and Biophysical Research Communications*.

[14] Bernardi S, Burns WC, Toffoli B (2012). Angiotensin-converting enzyme 2 regulates renal atrial natriuretic peptide through angiotensin-(1-7). *Clinical Science*.

[15] Ogawa Y, Mukoyama M, Yokoi H (2012). Natriuretic peptide receptor guanylyl cyclase-A protects podocytes from aldosterone-induced glomerular injury. *Journals of the American Society of Nephrology*.

[16] Arjamaa O, Nikinmaa M (2011). Hypoxia regulates the natriuretic peptide system. *International Journal of Physiology, Pathophysiology and Pharmacology*.

[17] Cavallero S, González GE, Puyó AM (2007). Atrial natriuretic peptide behaviour and myocyte hypertrophic profile in combined pressure and volume-induced cardiac hypertrophy. *Journal of Hypertension*.

[18] Puyó AM, Scaglione J, Auger S (2002). Atrial natriuretic factor as marker of myocardial compromise in Chagas disease. *Regulatory Peptides*.

[19] Zhu Q, Liu M, Han WQ, Li PL, Wang Z, Li N (2012). Overexpression of HIF Prolyl-Hydoxylase-2 transgene in the renal medulla induced a salt sensitive hypertension. *Journal of Cellular and Molecular Medicine*.

[20] Della Penna SL, Cao G, Fellet A (2012). Salt-induced downregulation of renal aquaporins is prevented by losartan. *Regulatory Peptides*.

[21] Wilcox CS, Pearlman A (2008). Chemistry and antihypertensive effects of tempol and other nitroxides. *Pharmacological Reviews*.

[22] Wärntges S, Gröne HJ, Capasso G, Lang F (2001). Cell volume regulatory mechanisms in progression of renal disease. *Journal of Nephrology*.

[23] Meng XM, Huang XR, Chung AC (2010). Smad2 protects against TGF-*β*/Smad3-mediated renal fibrosis. *Journal of the American Society of Nephrology*.

[24] Li P, Oparil S, Novak L (2007). ANP signaling inhibits TGF-*β*-induced Smad2 and Smad3 nuclear translocation and extracellular matrix expression in rat pulmonary arterial smooth muscle cells. *Journal of Applied Physiology*.

[25] Li P, Wang D, Lucas J (2008). Atrial natriuretic peptide inhibits transforming growth factor *β*-induced Smad signaling and myofibroblast transformation in mouse cardiac fibroblasts. *Circulation Research*.

[26] Rosón MI, Toblli JE, Della Penna SL (2006). Renal protective role of atrial natriuretic peptide in acute sodium overload-induced inflammatory response. *American Journal of Nephrology*.

[27] Gradin K, Hedner J, Hedner T, Towle AC, Pettersson A, Persson B (1987). Effects of chronic salt loading on plasma atrial natriuretic peptide (ANP) in the spontaneously hypertensive rat. *Acta Physiologica Scandinavica*.

[28] Graffe CC, Bech JN, Pedersen EB (2012). Effect of high and low sodium intake on urinary aquaporin-2 excretion in healthy humans. *The American Journal of Physiology*.

[29] Sagnella GA, Markandu ND, Buckley MG (1991). Atrial natriuretic peptides in essential hypertension: basal plasma levels and relationship to sodium balance. *Canadian Journal of Physiology and Pharmacology*.

[30] Lee KS, Kim SY, Han JH (2004). Different responses of atrial natriuretic peptide secretion and its receptor density to salt intake in rats. *Experimental Biology and Medicine*.

[31] Sun JZ, Chen SJ, Majid-Hasan E, Oparil S, Chen YF (2002). Dietary salt supplementation selectively downregulates NPR-C receptor expression in kidney independently of ANP. *The American Journal of Physiology*.

[32] Weinstein SW, Klose R, Szyjewicz J (1984). Proximal tubular Na, Cl, and HCO3 reabsorption and renal oxygen consumption. *The American Journal of Physiology*.

[33] Li N, Chen L, Yi F, Xia M, Li PL (2008). Salt-sensitive hypertension induced by decoy of transcription factor hypoxia-inducible factor-1*α* in the renal medulla. *Circulation Research*.

[34] Yang ZZ, Zhang AY, Yi FX, Li PL, Zou AP (2003). Redox regulation of HIF-1*α* levels and HO-1 expression in renal medullary interstitial cells. *The American Journal of Physiology*.

[35] Yang ZZ, Zou AP (2001). Transcriptional regulation of heme oxygenases by HIF-1*α* in renal medullary interstitial cells. *The American Journal of Physiology*.

